# Mitochondrial Haplogroups and Control Region Polymorphisms Are Not Associated with Prostate Cancer in Middle European Caucasians

**DOI:** 10.1371/journal.pone.0006370

**Published:** 2009-07-28

**Authors:** Edith E. Mueller, Waltraud Eder, Johannes A. Mayr, Bernhard Paulweber, Wolfgang Sperl, Wolfgang Horninger, Helmut Klocker, Barbara Kofler

**Affiliations:** 1 Department of Pediatrics, Paracelsus Medical University Salzburg, Salzburg, Austria; 2 Department of Internal Medicine, Paracelsus Medical University Salzburg, Salzburg, Austria; 3 Division of Experimental Urology, Department of Urology, Innsbruck Medical University, Innsbruck, Austria; Memorial Sloan-Kettering Cancer Center, United States of America

## Abstract

**Background:**

Besides being responsible for energy production in the cell, mitochondria are central players in apoptosis as well as the main source of harmful reactive oxygen species. Therefore, it can be hypothesised that sequence variation in the mitochondrial genome is a contributing factor to the etiology of diseases related to these different cellular events, including cancer. The aim of the present study was to assess the frequency of haplogroups and polymorphisms in the control region (CR) of mitochondrial DNA of peripheral blood mononuclear cells from patients with prostate carcinoma (n = 304) versus patients screened for prostate disease but found to be negative for cancer on biopsy (n = 278) in a Middle European population.

**Methodology/Principal Findings:**

The nine major European haplogroups and the CR polymorphisms were identified by means of primer extension analysis and DNA sequencing, respectively. We found that mitochondrial haplogroup frequencies and CR polymorphisms do not differ significantly between patients with or without prostate cancer, implying no impact of inherited mitochondrial DNA variation on predisposition to prostate carcinoma in a Middle European population.

**Conclusions/Significance:**

Our results contrast with a recent report claiming an association between mtDNA haplogroup U and prostate cancer in a North American population of caucasian descent.

## Introduction

Prostate cancer is a major cause of death in the developed world. It is the most frequent cancer among men in the United States and the second most common in the European Union [1], [2].

A shift in cellular energy production from oxidative phosphorylation in mitochondria to anaerobic glycolysis, called the Warburg effect, is a fundamental property of cancer cells. Due to the essential role of enzymes encoded by the mitochondrial DNA (mtDNA) in energy metabolism, alterations to the mitochondrial genome have been suspected to be able to cause a metabolic shift in tumors [3]. Furthermore, mitochondrial respiratory activity is associated with the generation of reactive oxygen species (ROS), which may contribute to the etiology and progression of cancer [4]. Because mitochondria play important roles in both ROS production and apoptosis, the conclusion that they could influence the occurrence of cancer is easily drawn.

Most eukaryotic cells contain hundreds of mitochondria which harbor numerous copies of mtDNA [5], [6]. The circular and double-stranded mitochondrial genome is around 16 kilobase pairs long and can be further divided into two main regions: the coding region and the control region. The coding region consists of 37 genes, 24 encoding components of the mitochondrial translational machinery and 13 genes providing essential subunits of the energy-generating enzymes of the oxidative phosphorylation (OXPHOS) pathway [5], [7]. The control region (CR), which is non-coding, contains two hypervariable regions (HVI and HVII) and a displacement (D-loop) region, and is involved in mtDNA replication and transcription [8].

The human population has accumulated a high number of mtDNA base substitutions along radiating maternal lineages, of which specific combinations constitute the so-called mitochondrial haplogroups [7], [9]. Nine distinct European mtDNA haplogroups (H, U, J, T, K, W, I, V, X) were defined by Torroni et al. [10], [11]. Although most mitochondrial polymorphisms are believed to be neutral, population-specific mtDNAs may be functionally different and exert varying influences on the outcomes of diseases [7], [12]–[14].

Mitochondrial haplogroups and polymorphisms have been found to be linked with carcinogenesis. For example, the mtDNA 10398A polymorphism is implicated in enhanced ROS production, and was found to be a risk factor for breast cancer and esophageal cancer in Indian patients, and for invasive breast cancer in African-American women [4], [15]. In addition, Asian haplogroup D was found to be associated with a higher risk of developing endometrial cancer in China [16]. In Caucasian populations, haplogroup K was associated with a significant increase, and haplogroup U a significant decrease, in breast cancer risk [17].

Booker et al. [18] reported an elevated frequency of haplogroup U in prostate carcinoma patients in a study of North American whites. This Caucasian haplogroup was correlated with a 2-fold risk of developing the disease [18].

We aimed to replicate the latter study on a Middle European population. Because associations of age-related diseases have been demonstrated not only with mitochondrial haplogroups, but also with single nucleotide polymorphisms (SNPs) in the CR [19], we also investigated sequence variations in HVI and HVII in our cohorts.

## Results

We assessed the nine major European haplogroups as well as CR polymorphisms in peripheral blood mononuclear cells of 582 Caucasian males, 304 of whom were diagnosed with prostate cancer; the remaining 278 were identified with elevated serum PSA levels but were histologically negative for cancer upon prostate biopsy and served as the control group. Clinical characteristics of the cancer patients and controls are shown in Table 1. All nine European haplogroups were observed in our cohort. The frequencies of mitochondrial haplogroups did not differ significantly between the patients with prostate cancer and the control subjects (Table 2). When mitochondrial haplogroup frequencies were compared between cancer patients with biopsy Gleason Score ≤6 to cancer patients with biopsy Gleason Score ≥7 also no significant differences were observed (Table 3).

**Table 1:** Characteristics of the study populations.

	Patients with prostate carcinoma	Patients with benign changes
	n = 304	n = 278
Mean (SD^1^) age at diagnosis (years)	70.3 (5.4)	66.1 (5.5)
Mean (SD^1^) PSA^2^ (ng/ml)	24.0 (125.6)	5.4 (4.0)
Prostate volume (g)	35.4 (21.0)	44.3 (33.4)
Biopsy defined as benign	0	100
Mean (SD^1^) Gleason Score	6.4 (1.3)	-
Gleason Score ≤6 (%)	55.9	-
Gleason Score ≥7 (%)	44.1	-

1 SD: Standard deviation.

2 PSA: Prostate specific antigen.

**Table 2:** Frequencies (%) of Caucasian mitochondrial haplogroups in cases and controls.

Haplogroup	Patients with prostate carcinoma	Patients with benign changes	P-Value^1^
	n = 304	n = 278	
H	39.1	36.0	0.430
U	16.1	16.9	0.798
J	8.6	9.0	0.851
T	12.8	13.6	0.765
K	7.9	5.4	0.228
W	0.7	1.8	0.267
V	2.0	1.4	0.754
I	1.6	2.9	0.315
X	2.3	1.1	0.344
Others^2^	8.9	11.9	0.236

1 P-Value: Pearson chi-square or Fisher's exact test.

2 Haplogroups that could not be assigned to one of the nine major European haplogroups by the SNP combination.

**Table 3:** Frequencies (%) of Caucasian mitochondrial haplogroups in prostate carcinoma patients with biopsy Gleason Score Values ≤6 and ≥7.

Haplogroup	Patients with prostate carcinoma and Gleason Score ≤6	Patients with prostate carcinoma and Gleason Score ≥7	P-Value^1^
	n = 170	n = 134	
H	41.8	35.8	0.292
U	15.3	17.2	0.660
J	8.2	9.0	0.824
T	12.4	13.4	0.780
K	7.6	8.2	0.857
W	0.0	1.5	0.193
V	1.2	3.0	0.411
I	1.8	1.5	1.000
X	2.3	2.2	1.000
Others^2^	9.4	8.2	0.714

1 P-Value: Pearson chi-square or Fisher's exact test.

2 Haplogroups that could not be assigned to one of the nine major European haplogroups by the SNP combination.

The mitochondrial CR was sequenced and analyzed between nucleotide positions 16145 and 530. Two hundred and nineteen polymorphisms were found among the 582 subjects, with 197 homoplasmic single base-pair exchanges, 10 single base-pair deletions and 5 single base-pair insertions when compared to the revised Cambridge Reference Sequence. Two samples presented the same 6 base-pair deletion of nucleotides 106 to 111. A CA-deletion at positions 514 and 515 occurred 40 times, and CA-insertions and CACA-insertions were also found at this site 64 times. CC-insertions occurred at positions 302 and 310, and heteroplasmy was found in one sample (A 16280 A/G). Of these 219 polymorphisms, 16 are not listed in the MITOMAP or Human Mitochondrial Genome Database (www.mitomap.org; www.genpat.uu.se/mtDB/). All polymorphisms and their frequencies in the control and cancer study groups are listed in Supplementary Table S1.

Two of the 219 polymorphisms - A 189 G, and T 310 C - were found to have a significantly lower frequency in the cancer patients compared to the control population (p<0.05) (Table 4, Supplementary Table S1). However, the significance was lost after correction for multiple comparisons (Bonferroni analysis), leading to a new required significance level of <0.00023. Table 4 lists the 35 polymorphisms with a frequency ≥5% in either the control or prostate cancer study group.

**Table 4:** Frequencies of control region polymorphisms greater than 5% in either the control or cancer cohort.

Polymorphism in mtDNA control region	Frequency in Control Cohort (%)	n^1^	Frequency in Cancer Cohort (%)	n^1^	P-Value^2^	Odds Ratio (95% CI^3^)
T 16172 C	3.60	10	5.26	16	0.331	
T 16189 C	13.31	37	15.13	46	0.530	
C 16192 T	5.76	16	6.58	20	0.680	
C 16223 T	8.63	24	6.58	20	0.349	
T 16224 C	7.55	21	9.54	29	0.393	
C 16256 T	6.83	19	5.59	17	0.534	
C 16270 T	9.35	26	8.88	27	0.844	
C 16294 T	14.39	40	13.82	42	0.843	
C 16296 T	8.63	24	5.26	16	0.108	
T 16304 C	12.95	36	8.22	25	0.063	
T 16311 C	18.71	52	17.11	52	0.615	
T 16356 C	5.04	14	3.29	10	0.290	
T 16362 C	11.51	32	7.57	23	0.104	
T 16519 C	63.67	177	65.13	198	0.713	
A 73 G	60.07	167	56.25	171	0.351	
T 146 C	8.99	25	9.21	28	0.927	
C 150 T	7.19	20	10.20	31	0.201	
T 152 C	21.58	60	18.75	57	0.394	
G 185 A	8.27	23	7.89	24	0.867	
A 189 G	5.76	16	1.97	6	0.017	0.330 (0.127–0.855)
T 195 C	17.27	48	14.47	44	0.356	
T 199 C	5.04	14	4.61	14	0.808	
T 204 C	7.19	20	4.93	15	0.252	
G 228 A	7.19	20	7.89	24	0.749	
A 263 G	98.92	275	99.34	302	0.674	
C 295 T	9.35	26	9.54	29	0.939	
A 302 C-Ins	48.92	136	43.75	133	0.211	
A 302 CC-Ins	13.31	37	13.49	41	0.950	
T 310 C-Ins	97.12	270	99.34	302	0.054	
C 462 T	8.63	24	9.21	28	0.807	
T 489 C	11.15	31	9.87	30	0.614	
C 497 T	2.88	8	5.92	18	0.076	
G 499 A	5.04	14	3.29	10	0.290	
G 513 CA-Ins	8.99	25	5.92	18	0.157	
514/515 Del	8.99	25	4.93	15	0.053	

1 n = Number of individuals with the respective polymorphism.

2 P-Value: Pearson chi-square or Fisher's exact test.

3 CI = Confidence interval.

## Discussion

The aim of the present study was to replicate the survey of Booker et al. [18], who performed a case-control study on 221 white men with prostate cancer and 246 white controls in North America and found mitochondrial haplogroup U to be overrepresented in the cancer group compared to the control group (OR: 1.95). In Middle European Caucasians we did not find significant differences between the haplogroup distributions in prostate cancer patients compared to the controls. Adjustment for multiple comparisons applied to the study of Booker et al. also led to loss of statistical significance [18]. The haplogroup distribution of the North American study closely resembles the distribution in our cancer patients, whereas the control group in the study of Booker et al. [18] is underrepresented for haplogroup U by about 45% compared to our control group (Table 5). When we compared the American case group to the Austrian control group no significant association of haplogroups to prostate cancer was detected.

**Table 5:** Comparison of haplogroup U frequencies and important characteristics in different mitochondrial haplogroup studies.

		Haplogroup U %	P-Value^1^	Mean (SD^2^) age	% male
Patients with benign changes (current study)	n = 278	16.9	-	66.1 (5.5)	100
Patients with prostate carcinoma (current study)	n = 304	16.1	0.798	70.3 (5.4)	100
Salzburg Atherosclerosis Prevention Program (SAPHIR), healthy subjects (Wiesbauer et al., [20])	n = 1527	15.5	0.540	51.5 (6.1)	64.7
Population study of West European Caucasians of the same geographical region (random sample) (Brandstätter et al., [21])	n = 277	18.8	0.566	n.a.	n.a.
Patients with prostate carcinoma (Booker et al., [18])	n = 221	16.7	0.961	56.7 (8.5)	100
Control population (Booker et al., [18])	n = 246	9.3	0.011	34.2 (16.6)	n.a.

1 P-Values shown represent the comparison of haplogroup U frequencies between the control population of the present study and the other listed cohorts (Pearson chi-square statistics).

2 SD: Standard deviation; n.a.: not available.

To exclude the possibility that having prostate problems in general – indicated by elevated PSA serum levels in our patient and control group - is associated with a higher haplogroup U frequency, we further assessed the haplogroup U frequency among participants in the SAPHIR study (Salzburg Atherosclerosis Prevention Program, as previously published [14], [20]). The frequencies of haplogroup U in our control and prostate cancer groups are not significantly different from those of these healthy SAPHIR subjects. A subgroup of this population, consisting only of men (n = 988, mean age 49 years), also has a frequency of haplogroup U similar to the prostate cancer group (data not shown). Furthermore, the frequency of haplogroup U among 277 random West European Caucasians studied by Brandstätter et al. [21] also is not significantly different from the frequency in our control group (Table 5). The subjects of that study were enrolled at the same University Hospital in Austria where our patient and control cohorts were recruted.

In mtDNA-related epidemiological studies the importance of appropriate control groups has been emphasized previously [22], [23]. There are several reasons that might explain the discrepant results. For example, regional variation, age or sex differences could influence the outcome of statistical calculations. Booker et al. [18] do not define whether their control population consisted entirely of men or also included women. Their control cohort consisted of organ donors, who in the United States must explicitly state their wish to become an organ donor; thus, depending on the propensities of different ethnic groups to donate organs, organ donor banks might exhibit different haplogroup distributions compared to the general population. The mean age of their controls was less than that of the patients with cancer. Age could have an impact on regional population variation, especially in a country such as the United States with a large immigrant population. Immigration patterns might have changed over a period of time, leading to different haplogroup distributions in different age groups of a population.

In another US study which sought to replicate the findings of Booker et al. [18] with respect to an association between haplogroup U and prostate cancer in white men, Canter et al. [24] reported haplogroup U percentages of 26.7% for their prostate cancer group (n = 71) and 11.7% for their control group (n = 128). A limitation of their study is the low number of prostate cancer patients. Moreover, their brief report does not give characteristics of the study participants or how they were selected.

It can not be excluded that geographical variation of haplogroup frequencies in control study populations are present between Austria and the USA.

In accordance with our results, a Korean study found no association between any mitochondrial haplogroups and prostate cancer [25]. The common set of 22 East Asian haplogroups was examined and no statistically significant difference in the distributions of mtDNA haplogroup frequencies was observed between the case (n = 139) and control groups (n = 122).

Comparing CR polymorphisms of cancer patients to controls we found two out of 219 statistically significant differences, which lost their significance after correction for multiple comparisons.

As we analyzed only mitochondrial haplogroups and control region polymorphisms, we cannot exclude the possibility that polymorphisms in the mitochondrial coding region, not included in the predefined haplogroup polymorphisms, could be associated with prostate cancer. Another limitation of our analysis is the low study power. Very large sample sizes would be required to reliably detect a modest difference in haplogroup frequencies between two groups [26].

Somatic mutations of mtDNA have been reported in a variety of cancers, including prostate carcinoma [27], [28]. In this study, only blood genomic DNA samples were available to us, and therefore no associations between somatic mutations/polymorphisms could be determined.

In conclusion, we did not find any mitochondrial haplogroups or CR polymorphisms to be associated with prostate cancer in an Austrian population.

## Materials and Methods

### Ethics Statement

The study was conducted according to the Austrian Gene Technology Act and complied with the Declaration of Helsinki. The study and the use of archive samples for the study was approved by the Ethics Committee of the Innsbruck Medical University (study UN 3174). Samples archived in 1995 to 2006 were used for the study. Written informed consent was obtained starting in 2001.

### Patients and control subjects

A total of 582 Caucasian subjects were analyzed; all were recruited from the Department of Urology of the Innsbruck Medical University. Patients and controls were enrolled in the Tyrolean early detection program for prostate cancer, which is based on regular PSA (prostate specific antigen) testing [29], [30]. The case group comprises 304 men aged 51 to 84 years, diagnosed with prostate cancer by histopathological evaluation according to World Health Organisation recommendations. As a disease control group, 278 men aged 46 to 80 years with benign results of prostate biopsy were recruited as previously described [29] (Table 1). For the disease control group a mean follow-up of 4.3 years after biopsy is available ranging from zero to 12 years, where no cancer was diagnosed.

### DNA isolation and mtDNA analysis

DNA was isolated from frozen peripheral blood mononuclear cells (PBMCs) using the AllPrep DNA/RNA Mini Kit 50 (Qiagen, Hilden, Germany). A hierarchical system for mtDNA haplogrouping that combines multiplex PCR amplification, multiplex single-base primer extension, and capillary-based electrophoretic separation for analyzing ten haplogroup-diagnostic mitochondrial SNPs (mtSNPs) was used to determine the haplogroup distribution of the most common European haplogroups, H, U, J, T, K, I, V, W and X, as described previously [20].

However, the following changes were made to this protocol: To remove primers and unincorporated deoxynucleotides from the PCR products, an ExoSAP-IT (USB, Cleveland, OH, USA) digest in a volume of 4.5 µl containing 0.5 µl ExoSAP and 2 µl PCR product was performed at 37°C for 60 min followed by enzyme inactivation at 80°C for 15 min. Multiplex primer extension reactions were carried out in a total volume of 5 µl containing 1 µl of SNP Start Master Mix (GenomeLab™ SNP Start Primer Extension Kit, Beckman Coulter, Fullerton, CA, USA), 1 µl of PCR product, and 1 µl of primer mix. Two SNP primers were changed to 8251r: (A)_15_GAGGGGGTGCTATAGGGTAAATACGGG and 16391r: (A)_6_TGATTTCACGGAGGATGGTGGTCAAGGGA. As an internal standard, fluorescently labeled primers (Biomers, Ulm, Germany) of lengths 15 and 60 bases were used (15 bases standard DY781: CACATGTCGGAGTCT, 60 bases standard DY781: TACAGTTCGTGCACACCGGCATCAGCTGTGTGCGAGAGTACTTACTATTGGTTGGCCAGA).

Haplogroups that could not be assigned to one of the nine major European haplogroups by the SNP combination were designated as “others”.

CR sequences were analyzed between nucleotide positions 16145 and 530, from a 1066-bp fragment. The fragment was amplified with primers 16098f: ACATTACTGCCAGCCACCATG and 638r: GGTGATGTGAGCCCGTCTAAAC. The PCR was performed in a volume of 30 µl containing 10× PCR buffer B (Solis Biodyne, Tartu, Estonia), 2.5 mM MgCl_2_, 333.3 pM of forward and reverse primer, 133.3 µM of each deoxynucleotide triphosphate (Invitrogen, Carlsbad, CA, USA) and 0.083 U of Hot Fire Polymerase (Solis Biodyne, Tartu, Estonia). Thermal cycling conditions were 95°C for 15 minutes, 35 cycles at 95°C for 30 seconds, 58°C for 30 seconds and 72°C for 2 minutes, and finally 37°C for 2 minutes.

PCR products were purified the same way as described for haplogroup analysis (ExoSAP-IT, USB, Cleveland, OH, USA). The fragment was sequenced using primers 16098f: ACATTACTGCCAGCCACCATG and 17f: CCCTATTAACCACTCACGGG. Sequencing was conducted using GenomeLab™ DTCS – Quick Start Kit (Beckman Coulter, Fullerton, CA, USA) in a volume of 10 µl containing 2 µl of the Master Mix and 500 pM of the appropriate primer. Thermal cycling conditions for sequencing were performed with: 30 cycles of denaturation at 96°C for 5 seconds, annealing at 50°C for 5 seconds and extension at 60°C for 4 minutes, followed by 25°C for 10 seconds. Samples were ethanol-precipitated and separated by capillary electrophoresis on a Beckman Coulter CEQ™ 2000 Genetic Analysis System.

### Statistical analysis

Frequencies of all mitochondrial haplogroups and CR polymorphisms in the prostate cancer patients and controls were tested for independency using Pearson chi-square statistics and Fisher's exact test as appropriate. Furthermore, in the same way, frequencies of mitochondrial haplogroups in patients with prostate carcinoma and biopsy Gleason Score ≤6 and patients with prostate carcinoma and biopsy Gleason Score ≥7 were tested. A p-value<0.05 was considered statistically significant. For analysis of CR polymorphisms significant p-values were corrected for multiple comparisons by Bonferroni analysis (required significance level = 0.05/number of comparisons), leading to a new required significance level of <0.00023 [number of comparisons = 219]. All analyses were performed using SPSS 15.0 student version (SPSS GmbH Software, 80339 Munich, Germany).

## Supporting Information

Table S1 Control region polymorphisms.
(0.21 MB DOC)

## References

[1] Gomez-Zaera M, Abril J, Gonzalez L, Aguilo F, Condom E (2006). Identification of somatic and germline mitochondrial DNA sequence variants in prostate cancer patients.. Mutat Res.

[2] Jemal A, Siegel R, Ward E, Hao Y, Xu J (2008). Cancer statistics, 2008.. CA Cancer J Clin.

[3] Warburg O (1956). On the origin of cancer cells.. Science.

[4] Canter JA, Kallianpur AR, Parl FF, Millikan RC (2005). Mitochondrial DNA G10398A polymorphism and invasive breast cancer in African-American women.. Cancer Res.

[5] Ballard JW, Whitlock MC (2004). The incomplete natural history of mitochondria.. Mol Ecol.

[6] Taanman JW (1999). The mitochondrial genome: structure, transcription, translation and replication.. Biochim Biophys Acta.

[7] Wallace DC, Brown MD, Lott MT (1999). Mitochondrial DNA variation in human evolution and disease.. Gene.

[8] Penta JS, Johnson FM, Wachsman JT, Copeland WC (2001). Mitochondrial DNA in human malignancy.. Mutat Res.

[9] Torroni A, Wallace DC (1994). Mitochondrial DNA variation in human populations and implications for detection of mitochondrial DNA mutations of pathological significance.. J Bioenerg Biomembr.

[10] Torroni A, Lott MT, Cabell MF, Chen YS, Lavergne L (1994). mtDNA and the origin of Caucasians: identification of ancient Caucasian-specific haplogroups, one of which is prone to a recurrent somatic duplication in the D-loop region.. Am J Hum Genet.

[11] Torroni A, Huoponen K, Francalacci P, Petrozzi M, Morelli L (1996). Classification of European mtDNAs from an analysis of three European populations.. Genetics.

[12] Brown MD, Torroni A, Reckord CL, Wallace DC (1995). Phylogenetic analysis of Leber's hereditary optic neuropathy mitochondrial DNA's indicates multiple independent occurrences of the common mutations.. Hum Mutat.

[13] Brown MD, Sun F, Wallace DC (1997). Clustering of Caucasian Leber hereditary optic neuropathy patients containing the 11778 or 14484 mutations on an mtDNA lineage.. Am J Hum Genet.

[14] Kofler B, Mueller EE, Eder W, Stanger O, Maier R (2009). Mitochondrial DNA haplogroup T is associated with coronary artery disease and diabetic retinopathy: a case control study.. BMC Med Genet.

[15] Darvishi K, Sharma S, Bhat AK, Rai E, Bamezai RN (2007). Mitochondrial DNA G10398A polymorphism imparts maternal Haplogroup N a risk for breast and esophageal cancer.. Cancer Lett.

[16] Xu L, Hu Y, Chen B, Tang W, Han X (2006). Mitochondrial polymorphisms as risk factors for endometrial cancer in southwest China.. Int J Gynecol Cancer.

[17] Bai RK, Leal SM, Covarrubias D, Liu A, Wong LJ (2007). Mitochondrial genetic background modifies breast cancer risk.. Cancer Res.

[18] Booker LM, Habermacher GM, Jessie BC, Sun QC, Baumann AK (2006). North American white mitochondrial haplogroups in prostate and renal cancer.. J Urol.

[19] Abril J, de Heredia ML, Gonzalez L, Cleries R, Nadal M (2008). Altered expression of 12S/MT-RNR1, MT-CO2/COX2, and MT-ATP6 mitochondrial genes in prostate cancer.. Prostate.

[20] Wiesbauer M, Meierhofer D, Mayr JA, Sperl W, Paulweber B (2006). Multiplex primer extension analysis for rapid detection of major European mitochondrial haplogroups.. Electrophoresis.

[21] Brandstatter A, Parsons TJ, Parson W (2003). Rapid screening of mtDNA coding region SNPs for the identification of west European Caucasian haplogroups.. Int J Legal Med.

[22] Pereira L, Goncalves J, Goios A, Rocha T, Amorim A (2005). Human mtDNA haplogroups and reduced male fertility: real association or hidden population substructuring.. Int J Androl.

[23] Dubut V, Chollet L, Murail P, Cartault F, Beraud-Colomb E (2004). mtDNA polymorphisms in five French groups: importance of regional sampling.. Eur J Hum Genet.

[24] Canter JA, Kallianpur AR, Fowke JH (2006). Re: North American white mitochondrial haplogroups in prostate and renal cancer.. J Urol.

[25] Kim W, Yoo TK, Shin DJ, Rho HW, Jin HJ (2008). Mitochondrial DNA haplogroup analysis reveals no association between the common genetic lineages and prostate cancer in the Korean population.. PLoS ONE.

[26] Samuels DC, Carothers AD, Horton R, Chinnery PF (2006). The power to detect disease associations with mitochondrial DNA haplogroups.. Am J Hum Genet.

[27] Chen JZ, Gokden N, Greene GF, Mukunyadzi P, Kadlubar FF (2002). Extensive somatic mitochondrial mutations in primary prostate cancer using laser capture microdissection.. Cancer Res.

[28] Taylor RW, Turnbull DM (2005). Mitochondrial DNA mutations in human disease.. Nat Rev Genet.

[29] Oberaigner W, Horninger W, Klocker H, Schonitzer D, Stuhlinger W (2006). Reduction of prostate cancer mortality in Tyrol, Austria, after introduction of prostate-specific antigen testing.. Am J Epidemiol.

[30] Bartsch G, Horninger W, Klocker H, Pelzer A, Bektic J (2008). Tyrol Prostate Cancer Demonstration Project: early detection, treatment, outcome, incidence and mortality.. BJU Int.

