# Supplementary material for: Mitochondrial Haplogroups and Control Region Polymorphisms Are Not Associated with Prostate Cancer in Middle European Caucasians
Source: PLoS One. 2009 Jul 28;4(7):e6370. doi: 10.1371/journal.pone.0006370 (PMC2712094; doi:10.1371/journal.pone.0006370)
Supplement: Table S1 — Control region polymorphisms. (0.21 MB DOC) [file pone.0006370.s001.doc]

**Table S1.** Control region polymorphisms.

| Polymorphism in mtDNA control region | Frequency (%) in Control Cohort | n^1^ | Frequency (%) in Cancer Cohort | n^1^ | P-Value^2^ | Odds Ratio (95% CI^3^) |
| --- | --- | --- | --- | --- | --- | --- |
| G 16145 A | 1.80 | 5 | 2.30 | 7 | 0.669 |  |
| A 16146 G | 0.00 | 0 | 0.33 | 1 | 1.000 |  |
| C 16147 A | 0.00 | 0 | 1.64 | 5 | 0.063 |  |
| C 16147 T | 0.36 | 1 | 0.00 | 0 | 0.478 |  |
| C 16148 T | 1.44 | 4 | 0.66 | 2 | 0.432 |  |
| G 16153 A | 1.08 | 3 | 1.64 | 5 | 0.727 |  |
| T 16154 C | 0.00 | 0 | 0.98 | 3 | 0.250 |  |
| A 16162 G | 1.08 | 3 | 1.97 | 6 | 0.508 |  |
| A 16162 Del | 0.00 | 0 | 0.66 | 2 | 0.500 |  |
| A 16163 G | 2.52 | 7 | 2.96 | 9 | 0.744 |  |
| C 16167 T | 0.36 | 1 | 0.00 | 0 | 0.478 |  |
| C 16168 T | 0.72 | 2 | 1.32 | 4 | 0.688 |  |
| C 16169 T | 1.08 | 3 | 0.66 | 2 | 0.674 |  |
| A 16170 G | 0.36 | 1 | 0.33 | 1 | 1.000 |  |
| T 16172 C | 3.60 | 10 | 5.26 | 16 | 0.331 |  |
| C 16173 T | 0.36 | 1 | 0.33 | 1 | 1.000 |  |
| C 16179 T | 0.36 | 1 | 1.32 | 4 | 0.375 |  |
| A 16181 G | 0.36 | 1 | 0.00 | 0 | 0.478 |  |
| A 16182 C | 2.88 | 8 | 2.30 | 7 | 0.662 |  |
| A 16183 C | 4.68 | 13 | 4.28 | 13 | 0.816 |  |
| A 16183 Del | 0.00 | 0 | 0.33 | 1 | 1.000 |  |
| C 16184 T | 0.72 | 2 | 0.66 | 2 | 1.000 |  |
| C 16186 T | 2.52 | 7 | 2.63 | 8 | 0.931 |  |
| C 16187 T | 0.00 | 0 | 0.33 | 1 | 1.000 |  |
| T 16189 A | 1.08 | 3 | 0.00 | 0 | 0.108 |  |
| T 16189 C | 13.31 | 37 | 15.13 | 46 | 0.530 |  |
| T 16189 Del | 0.72 | 2 | 0.00 | 0 | 0.228 |  |
| T 16189 C-Ins | 0.00 | 0 | 0.33 | 1 | 1.000 |  |
| C 16192 T | 5.76 | 16 | 6.58 | 20 | 0.680 |  |
| C 16193 T | 1.80 | 5 | 0.33 | 1 | 0.109 |  |
| C 16201 T | 0.72 | 2 | 0.99 | 3 | 1.000 |  |
| T 16209 C | 0.72 | 2 | 0.99 | 3 | 1.000 |  |
| A 16212 G | 0.00 | 0 | 0.33 | 1 | 1.000 |  |
| G 16213 A | 1.80 | 5 | 1.32 | 4 | 0.743 |  |
| C 16214 G^4^ | 0.36 | 1 | 0.00 | 0 | 0.478 |  |
| A 16216 G | 0.36 | 1 | 0.00 | 0 | 0.478 |  |
| C 16218 T | 0.36 | 1 | 0.66 | 2 | 1.000 |  |
| A 16219 G | 0.36 | 1 | 0.33 | 1 | 1.000 |  |
| C 16221 T | 0.36 | 1 | 0.99 | 3 | 0.625 |  |
| C 16222 T | 1.44 | 4 | 1.97 | 6 | 0.754 |  |
| C 16223 T | 8.63 | 24 | 6.58 | 20 | 0.349 |  |
| T 16224 C | 7.55 | 21 | 9.54 | 29 | 0.393 |  |
| A 16227 C^4^ | 0.36 | 1 | 0.00 | 0 | 0.478 |  |
| T 16231 C | 0.72 | 2 | 0.66 | 2 | 1.000 |  |
| A 16233 G | 0.36 | 1 | 0.00 | 0 | 0.478 |  |
| C 16234 T | 1.80 | 5 | 0.99 | 3 | 0.489 |  |
| A 16235 G | 0.36 | 1 | 0.99 | 3 | 0.625 |  |
| C 16239 T | 0.00 | 0 | 0.33 | 1 | 1.000 |  |
| A 16240 G | 0.36 | 1 | 0.00 | 0 | 0.478 |  |
| C 16242 T | 0.36 | 1 | 0.33 | 1 | 1.000 |  |
| C 16248 T | 1.44 | 4 | 3.95 | 12 | 0.064 |  |
| T 16249 C | 0.72 | 2 | 0.33 | 1 | 0.608 |  |
| G 16255 A | 0.36 | 1 | 0.66 | 2 | 1.000 |  |
| C 16256 T | 6.83 | 19 | 5.59 | 17 | 0.534 |  |
| C 16256 Del | 0.00 | 0 | 0.33 | 1 | 1.000 |  |
| A 16258 C | 0.36 | 1 | 0.33 | 1 | 1.000 |  |
| C 16260 T | 1.44 | 4 | 0.66 | 2 | 0.432 |  |
| C 16261 T | 2.52 | 7 | 4.61 | 14 | 0.177 |  |
| C 16262 T | 0.36 | 1 | 0.00 | 0 | 0.478 |  |
| T 16263 C | 1.80 | 5 | 0.66 | 2 | 0.267 |  |
| C 16264 T | 0.00 | 0 | 0.33 | 1 | 1.000 |  |
| A 16265 G | 2.16 | 6 | 2.30 | 7 | 0.906 |  |
| C 16266 T | 2.88 | 8 | 1.64 | 5 | 0.315 |  |
| C 16270 T | 9.35 | 26 | 8.88 | 27 | 0.844 |  |
| T 16271 C | 0.36 | 1 | 0.00 | 0 | 0.478 |  |
| G 16274 A | 1.80 | 5 | 0.33 | 1 | 0.109 |  |
| C 16278 T | 2.52 | 7 | 2.30 | 7 | 0.866 |  |
| A 16280 A/G^4^ | 0.36 | 1 | 0.00 | 0 | 0.478 |  |
| C 16287 A | 0.00 | 0 | 0.33 | 1 | 1.000 |  |
| C 16287 T | 0.36 | 1 | 0.33 | 1 | 1.000 |  |
| A 16289 G | 0.00 | 0 | 0.33 | 1 | 1.000 |  |
| C 16290 T | 1.44 | 4 | 0.33 | 1 | 0.198 |  |
| C 16291 A^4^ | 0.00 | 0 | 0.33 | 1 | 1.000 |  |
| C 16291 T | 1.44 | 4 | 2.30 | 7 | 0.445 |  |
| C 16292 T | 1.80 | 5 | 2.30 | 7 | 0.669 |  |
| A 16293 G | 1.80 | 5 | 1.64 | 5 | 1.000 |  |
| C 16294 T | 14.39 | 40 | 13.82 | 42 | 0.843 |  |
| C 16295 T | 0.36 | 1 | 0.33 | 1 | 1.000 |  |
| C 16296 T | 8.63 | 24 | 5.26 | 16 | 0.108 |  |
| T 16298 C | 3.60 | 10 | 4.28 | 13 | 0.674 |  |
| A 16300 G | 0.36 | 1 | 0.00 | 0 | 0.478 |  |
| C 16301 T | 0.36 | 1 | 0.00 | 0 | 0.478 |  |
| G 16303 A | 0.00 | 0 | 0.33 | 1 | 1.000 |  |
| T 16304 C | 12.95 | 36 | 8.22 | 25 | 0.063 |  |
| T 16311 C | 18.71 | 52 | 17.11 | 52 | 0.615 |  |
| A 16316 G | 0.00 | 0 | 0.33 | 1 | 1.000 |  |
| A 16318 T | 0.36 | 1 | 0.00 | 0 | 0.478 |  |
| G 16319 A | 2.88 | 8 | 1.64 | 5 | 0.315 |  |
| C 16320 T | 0.36 | 1 | 1.64 | 5 | 0.219 |  |
| T 16324 C | 0.36 | 1 | 0.00 | 0 | 0.478 |  |
| T 16325 C | 0.36 | 1 | 0.99 | 3 | 0.625 |  |
| C 16327 T | 0.72 | 2 | 0.66 | 2 | 1.000 |  |
| T 16342 C | 0.36 | 1 | 0.33 | 1 | 1.000 |  |
| A 16343 G | 1.44 | 4 | 0.99 | 3 | 0.714 |  |
| C 16344 T | 0.36 | 1 | 0.00 | 0 | 0.478 |  |
| T 16352 C | 0.36 | 1 | 0.00 | 0 | 0.478 |  |
| C 16354 A | 0.00 | 0 | 0.33 | 1 | 1.000 |  |
| C 16354 T | 1.08 | 3 | 1.97 | 6 | 0.508 |  |
| C 16355 G^4^ | 0.36 | 1 | 0.00 | 0 | 0.478 |  |
| C 16355 T | 0.36 | 1 | 1.97 | 6 | 0.125 |  |
| T 16356 C | 5.04 | 14 | 3.29 | 10 | 0.290 |  |
| T 16357 C | 0.36 | 1 | 0.33 | 1 | 1.000 |  |
| T 16359 C | 0.36 | 1 | 0.00 | 0 | 0.478 |  |
| T 16362 C | 11.51 | 32 | 7.57 | 23 | 0.104 |  |
| C 16365 T | 0.00 | 0 | 0.33 | 1 | 1.000 |  |
| T 16368 C | 0.00 | 0 | 0.33 | 1 | 1.000 |  |
| G 16384 A-Ins^4^ | 0.00 | 0 | 0.66 | 2 | 0.500 |  |
| G 16388 Del^4^ | 0.00 | 0 | 0.33 | 1 | 1.000 |  |
| G 16390 A | 0.72 | 2 | 2.30 | 7 | 0.180 |  |
| G 16391 A | 2.88 | 8 | 1.64 | 5 | 0.315 |  |
| G 16398 A | 0.00 | 0 | 1.32 | 4 | 0.125 |  |
| A 16399 G | 4.68 | 13 | 3.62 | 11 | 0.521 |  |
| C 16400 T | 0.36 | 1 | 0.66 | 2 | 1.000 |  |
| G 16438 A | 0.00 | 0 | 0.33 | 1 | 1.000 |  |
| A 16463 G | 0.36 | 1 | 0.00 | 0 | 0.478 |  |
| C 16465 T | 0.36 | 1 | 0.00 | 0 | 0.478 |  |
| G 16474 C | 0.00 | 0 | 0.33 | 1 | 1.000 |  |
| A 16482 G | 1.44 | 4 | 0.66 | 2 | 0.432 |  |
| A 16497 G | 0.72 | 2 | 0.00 | 0 | 0.228 |  |
| T 16502 C^4^ | 0.00 | 0 | 0.33 | 1 | 1.000 |  |
| T 16519 C | 63.67 | 177 | 65.13 | 198 | 0.713 |  |
| A 16524 G | 0.00 | 0 | 0.33 | 1 | 1.000 |  |
| G 16526 A | 3.24 | 9 | 2.30 | 7 | 0.491 |  |
| C 16527 T | 0.00 | 0 | 0.33 | 1 | 1.000 |  |
| A 16 T^4^ | 0.00 | 0 | 0.33 | 1 | 1.000 |  |
| C 41 T | 0.36 | 1 | 0.00 | 0 | 0.478 |  |
| T 55 C | 0.36 | 1 | 0.66 | 2 | 1.000 |  |
| T 57 C | 0.36 | 1 | 0.66 | 2 | 1.000 |  |
| T 58 C | 0.36 | 1 | 0.33 | 1 | 1.000 |  |
| C 64 T | 1.08 | 3 | 0.66 | 2 | 0.674 |  |
| T 65 G^4^ | 0.00 | 0 | 0.33 | 1 | 1.000 |  |
| T 72 C | 2.52 | 7 | 2.63 | 8 | 0.931 |  |
| A 73 G | 60.07 | 167 | 56.25 | 171 | 0.351 |  |
| A 93 G | 0.72 | 2 | 1.32 | 4 | 0.688 |  |
| G 94 A | 0.36 | 1 | 0.33 | 1 | 1.000 |  |
| GGAGCA 106-111 Del | 0.36 | 1 | 0.33 | 1 | 1.000 |  |
| C 114 T | 0.72 | 2 | 0.66 | 2 | 1.000 |  |
| T 119 C | 0.36 | 1 | 0.00 | 0 | 0.478 |  |
| G 143 A | 0.72 | 2 | 0.33 | 1 | 0.608 |  |
| T 146 C | 8.99 | 25 | 9.21 | 28 | 0.927 |  |
| C 150 T | 7.19 | 20 | 10.20 | 31 | 0.201 |  |
| C 151 T | 2.52 | 7 | 0.99 | 3 | 0.206 |  |
| T 152 C | 21.58 | 60 | 18.75 | 57 | 0.394 |  |
| A 153 G | 2.16 | 6 | 1.64 | 5 | 0.650 |  |
| C 182 T | 0.36 | 1 | 0.99 | 3 | 0.625 |  |
| G 185 A | 8.27 | 23 | 7.89 | 24 | 0.867 |  |
| C 186 A | 0.36 | 1 | 0.00 | 0 | 0.478 |  |
| A 188 G | 1.08 | 3 | 0.66 | 2 | 0.674 |  |
| A 189 G | 5.76 | 16 | 1.97 | 6 | 0.017 | 0.330 (0.127-0.855) |
| C 194 T | 0.72 | 2 | 0.33 | 1 | 0.608 |  |
| T 195 C | 17.27 | 48 | 14.47 | 44 | 0.356 |  |
| C 198 T | 0.36 | 1 | 0.00 | 0 | 0.478 |  |
| T 199 C | 5.04 | 14 | 4.61 | 14 | 0.808 |  |
| A 200 G | 1.80 | 5 | 3.29 | 10 | 0.257 |  |
| G 203 A | 0.36 | 1 | 0.00 | 0 | 0.478 |  |
| T 204 C | 7.19 | 20 | 4.93 | 15 | 0.252 |  |
| G 207 A | 4.32 | 12 | 3.29 | 10 | 0.516 |  |
| A 215 G | 0.36 | 1 | 0.33 | 1 | 1.000 |  |
| T 217 C | 1.80 | 5 | 2.96 | 9 | 0.361 |  |
| G 225 A | 0.72 | 2 | 0.66 | 2 | 1.000 |  |
| T 226 C | 0.36 | 1 | 0.33 | 1 | 1.000 |  |
| A 227 G | 0.36 | 1 | 0.33 | 1 | 1.000 |  |
| G 228 A | 7.19 | 20 | 7.89 | 24 | 0.749 |  |
| G 228 T | 0.00 | 0 | 0.33 | 1 | 1.000 |  |
| A 234 G | 0.00 | 0 | 0.33 | 1 | 1.000 |  |
| A 235 G | 1.08 | 3 | 0.99 | 3 | 1.000 |  |
| T 239 C | 0.72 | 2 | 0.99 | 3 | 1.000 |  |
| C 242 T | 1.80 | 5 | 1.64 | 5 | 1.000 |  |
| G 247 A | 0.72 | 2 | 0.33 | 1 | 0.608 |  |
| A 248 Del | 0.00 | 0 | 0.33 | 1 | 1.000 |  |
| T 250 C | 3.24 | 9 | 1.97 | 6 | 0.337 |  |
| T 252 C | 0.00 | 0 | 0.66 | 2 | 0.500 |  |
| A 257 G | 0.36 | 1 | 0.99 | 3 | 0.625 |  |
| A 259 G | 0.36 | 1 | 0.33 | 1 | 1.000 |  |
| A 263 G | 98.92 | 275 | 99.34 | 302 | 0.674 |  |
| T 282 C | 0.00 | 0 | 0.33 | 1 | 1.000 |  |
| T 293 C | 0.00 | 0 | 0.99 | 3 | 0.250 |  |
| C 295 A | 0.36 | 1 | 0.00 | 0 | 0.478 |  |
| C 295 T | 9.35 | 26 | 9.54 | 29 | 0.939 |  |
| C 296 T | 0.36 | 1 | 0.00 | 0 | 0.478 |  |
| C 298 Del | 0.36 | 1 | 0.00 | 0 | 0.478 |  |
| A 302 C-Ins | 48.92 | 136 | 43.75 | 133 | 0.211 |  |
| A 302 CC-Ins | 13.31 | 37 | 13.49 | 41 | 0.950 |  |
| C 309 T-Ins | 0.36 | 1 | 0.33 | 1 | 1.000 |  |
| T 310 C | 2.88 | 8 | 0.33 | 1 | 0.016 | 0.111 (0.014-0.896) |
| T 310 C-Ins | 97.12 | 270 | 99.34 | 302 | 0.054 |  |
| T 310 CC-Ins | 0.00 | 0 | 0.33 | 1 | 1.000 |  |
| G 316 A | 0.36 | 1 | 0.00 | 0 | 0.478 |  |
| T 319 C | 0.36 | 1 | 0.00 | 0 | 0.478 |  |
| T 321 C | 0.36 | 1 | 0.00 | 0 | 0.478 |  |
| C 324 Del^4^ | 0.00 | 0 | 0.33 | 1 | 1.000 |  |
| C 327 T | 0.36 | 1 | 0.33 | 1 | 1.000 |  |
| C 338 T | 0.00 | 0 | 0.33 | 1 | 1.000 |  |
| C 340 T | 1.80 | 5 | 2.63 | 8 | 0.497 |  |
| A 385 G | 0.00 | 0 | 0.99 | 3 | 0.250 |  |
| A 390 G | 0.72 | 2 | 1.32 | 4 | 0.688 |  |
| T 392 A^4^ | 0.36 | 1 | 0.00 | 0 | 0.478 |  |
| T 408 A | 1.44 | 4 | 0.00 | 0 | 0.051 |  |
| A 419 G^4^ | 0.36 | 1 | 0.00 | 0 | 0.478 |  |
| C 438 T^4^ | 0.00 | 0 | 0.33 | 1 | 1.000 |  |
| T 452 Del | 0.72 | 2 | 0.99 | 3 | 1.000 |  |
| T 453 A^4^ | 0.36 | 1 | 0.00 | 0 | 0.478 |  |
| C 456 T | 4.32 | 12 | 2.63 | 8 | 0.265 |  |
| C 458 T | 0.00 | 0 | 0.33 | 1 | 1.000 |  |
| C 462 T | 8.63 | 24 | 9.21 | 28 | 0.807 |  |
| C 469 A | 0.00 | 0 | 0.33 | 1 | 1.000 |  |
| T 477 C | 2.52 | 7 | 1.64 | 5 | 0.459 |  |
| T 482 C | 2.88 | 8 | 1.32 | 4 | 0.185 |  |
| T 489 C | 11.15 | 31 | 9.87 | 30 | 0.614 |  |
| A 492 G^4^ | 0.00 | 0 | 0.33 | 1 | 1.000 |  |
| C 494 Del | 0.00 | 0 | 0.99 | 3 | 0.250 |  |
| C 497 T | 2.88 | 8 | 5.92 | 18 | 0.076 |  |
| G 499 A | 5.04 | 14 | 3.29 | 10 | 0.290 |  |
| T 507 C | 0.00 | 0 | 0.33 | 1 | 1.000 |  |
| A 508 G | 1.80 | 5 | 3.29 | 10 | 0.257 |  |
| G 513 A | 0.36 | 1 | 0.33 | 1 | 1.000 |  |
| G 513 CA-Ins | 8.99 | 25 | 5.92 | 18 | 0.157 |  |
| G 513 CACA-Ins | 3.96 | 11 | 3.29 | 10 | 0.666 |  |
| CA 514/515 Del | 8.99 | 25 | 4.93 | 15 | 0.053 |  |

^1^ n = Number of individuals with the respective polymorphism

^2^ P-Value: Pearson chi-square or Fisher’s exact test, respectively

^3^ CI = Confidence Interval

^4^ Polymorphisms not listed in the MITOMAP and Human Mitochondrial Genome
 Database
